# Five years of safety profile of bevacizumab: an analysis of real-world pharmacovigilance and randomized clinical trials

**DOI:** 10.1186/s40780-023-00314-w

**Published:** 2024-01-02

**Authors:** Li Wang, Yibo Fei, Han Qu, Haiyang Zhang, Yuanyuan Wang, Zhenghua Wu, Guorong Fan

**Affiliations:** 1 School of Pharmacy, Shanghai Jiao Tong University, Shanghai, 200240 China; 2 Department of Clinical Pharmacy, Shanghai General Hospital, Shanghai Jiaotong University, School of Medicine, Shanghai, 200080 China; 3 School of Pharmacy, Chongqing Medical University, Chongqing, 400016 China; 4 Department of Pharmacy, Shanghai Tenth People’s Hospital, Tongji University School of Medicine, Shanghai, 200072 China

**Keywords:** Bevacizumab, Safety, FDA adverse event reporting system, Pharmacovigilance, Meta-analysis

## Abstract

**Objective:**

Bevacizumab is a monoclonal antibody against vascular endothelial growth factor. It has a wide range of clinical applications in various cancers and retinal diseases. The drugs entered the Chinese market by a large margin in 2017, and the user population changed to some extent. This study reevaluated the safety of bevacizumab through an analysis of the World Pharmacovigilance database (Food and Drug Administration Open Vigil 2.1) in conjunction with a comprehensive meta-analysis of RCTs.

**Methods:**

Real-world pharmacovigilance data originating from case reports were mined using Open Vigil and coded at the preferred term (PT) level using the Standardized MedDRA Query. Proportional reporting ratios (PRR) and reporting odds ratios (ROR) were used to detect safety signals. Eligible items were screened by searching PubMed, Wanfang, and Web of Science, and data were extracted for systematic review and meta-analysis using RevMan 5.4 software.

**Results:**

Analysis of the drug pharmacovigilance database revealed that the most significant PRRs were limb decortication syndrome (PRR = 2926), stomal varices (PRR = 549), anastomotic (PRR = 457) and ureteral fistula (PRR = 406). Most safety signals at the PT level emerged as various types of injuries, toxicities, operational complications, systemic diseases, various reactions at the administration site, hematological and lymphatic disorders, and gastrointestinal disorders. Adverse reactions such as nasal septal perforation (PRR = 47.502), necrotizing fasciitis (PRR = 20.261), and hypertensive encephalopathy (PRR = 18.288) listed as rare in drug specifications should not be ignored with a high signal in the real world. A total of 8 randomized controlled trials (RCTs) were included in the meta-analysis, and the overall risk of adverse reactions following bevacizumab administration was relatively low, indicating a good safety profile (HR = 1.19, 95% CI:0.85 ~ 1.65, *p* = 0.32).

**Conclusion:**

The frequent adverse reactions of bevacizumab occurring in the real world are consistent with the data provided in RCTs and drug specifications. However, adverse reactions such as nasal septum perforation, necrotizing fasciitis, hypertensive encephalopathy and so on, listed as rare in drug specifications, may have a high signal of correlation in the real world, which all requires active monitoring and timely adjustment of bevacizumab posology during its clinical use.

**Supplementary Information:**

The online version contains supplementary material available at 10.1186/s40780-023-00314-w.

## Introduction

Bevacizumab belongs to a class of drugs that target vascular endothelial growth factors. Vascular endothelial growth factor (VEGF), also known as vascular permeability factor (VPF), is a growth factor with a highly specific mitogenic and anti-apoptotic effect on endothelial cells. It is involved in developing and progressing many angio-genesis-dependent diseases including cancer, certain inflammatory diseases, and diabetic retinopathy. VEGF is overexpressed in various types of cancers and is associated with lower recurrence-free and overall survival rates [1]. Besides inducing endothelial cell proliferation, it has pro-inflammatory effects by increasing endothelial cell permeability and determining vascular leakage. Thus, VEGF is involved in blood-retinal barrier disruption and retinal neovascularization, making the retina highly susceptible to a range of pathologies such as hemorrhage and exudative retinal detachment [2, 3]. Bevacizumab is a monoclonal antibody that targets VEGF. It blocks all isoforms of VEGF- A [4] and has shown clinical efficacy in a variety of cancers, including non-small cell lung cancer (NSCLC), advanced cervical cancer, glioblastoma, ovarian cancer and colorectal cancer [1, 5]. In addition to the authorized indications, it has also shown good efficacy in the treatment of macular edema and renal cell carcinoma [2, 6]. Thus, bevacizumab has a wide range of clinical applications, and its clinical effectiveness has been proven over time. However, many adverse reactions have been reported, some of which are tolerable, some that affect the quality of life of patients and require medical intervention, and some so severe that they can lead to the discontinuation of the drug or even death. Its most frequent adverse effects (AEs) include hypertension, fatigue, diarrhea and abdominal pain, and the more severe include gastrointestinal perforation, hemorrhage, and arterial thrombosis [7, 8]. However, most of these data are from clinical trials, and real-world data are still limited. before 2017, the price of bevacizumab in China was basically around 5,100 yuan per unit. Although the efficacy was precise, the high price of bevacizumab led to low accessibility of the drug for Chinese patients. At the end of 2017, the drug was significantly reduced in price through Chinese national negotiations and entered the medical insurance catalog with a price reduction of 60% or more, further expanding the user population. The willingness of patients to use drugs that were previously less used for price reasons will increase due to their positive efficacy, broad indications and significant price reductions. Drug companies compensate for the impact of price on sales revenue by exchanging price for volume while enhancing the accessibility of drugs. This may have biased the safety in-formation of the drug. For this reason, we have re-evaluated the safety of bevacizumab in all populations where it has been used since then Therefore, we sought to evaluate the safety of bevacizumab using randomized clinical trials and the Food and Drug Administration Adverse Event Re-Reporting System. Data mining techniques, such as signal detection algorithms, were used to explore the (FAERS) database and analyze the large amount of cumulative data derived from case reports to identify potential as-sociations between bevacizumab and AEs. The information in FAERS changes daily and the number of cases may increase or decrease. Therefore, information obtained from the website may also change over time. Many factors (e.g. product launch cycle, region, and underreporting) can affect case reporting, which can bias the initial safety assessment, such as overlooking the safety of some common adverse reactions. At present, the international methods used for ADR signal mining are mainly divided into frequency count method and Bayesian method. In this study, ROR and PRR in the frequency count method are used for data detection. The reporting probability (ROR) method was first proposed by the Dutch Center for Pharmacovigilance. ROR allows estimation of relative risk and focuses attention on which individuals or reports should be included or excluded from the control group, thus allowing for more effective elimination of bias. The proportional reporting rate (PRR) method was proposed by Evans et al. in 1998 to generate a positive signal [9]. the PRR value is related to the proportion of spontaneous reports of a drug associated with a specific adverse outcome divided by the corresponding proportion for all or several other drugs. This approach is similar to the construction of the proportional mortality rate (PMR) [10], an old epidemiological indicator that is calculated by death registries. This study is a systematic assessment of drug safety through real-world pharmacovigilance, centered on clinically relevant adverse event information, combined with a meta-analysis of clinical trials to complement and validate each other. However, the FAERS database also has some limitations. It is a spontaneous reporting system that relies on voluntary reporting by healthcare stakeholders, so there are problems of duplication, omissions, and missing information in the reporting of AEs, and it cannot completely exclude the effects of other drugs on a particularly adverse reaction. The information provided by this study adds to the information given in the drug specifications for improving the clinical use of bevacizumab. It is important to note that disproportionality analyses are indeed hypothetical and can only provide a preliminary statistical exploration of the possible adverse effects of a drug. More reliable conclusions require subsequent more rigorous experimental validation.

## Materials and methods

### Pharmacovigilance study

#### Data sources and processing

The data for this study were extracted from the public release of the FAERS data-base using OpenVigil FDA. OpenVigil FDA, a novel web-based pharmacovigilance analysis tool which uses the OpenVigil FDA online interface of the Food and Drug Administration (FDA) to access U.S. American and international pharmacovigilance data from the Adverse Event Reporting System (AERS). OpenVigil FDA provides disproportionality analyses to (i) identify the drug most likely evoking a new adverse event, (ii) compare two drugs concerning their safety profile, (iii) check arbitrary combinations of two drugs for unknown drug-drug interactions and (iv) enhance the relevance of results by identifying confounding factors and eliminating them using background correction. Pharmacovigilance is an open-world problem, i.e., the recorded data are from a subset of the entire population. This implies that any findings are useful for hypothesis generation only. In principle, pharmacovigilance cannot prove anything. Strict statisticians will find the analyses proposed above to be deceptive. However, this approach is the best the current health care system can actually provide. Common clinical practice aims are minimizing potential damage to the patient wherever possible. Any signals found with OpenVigil FDA, although somewhat imperfect, can be used to stimulate further research on pharmacokinetics and -dynamics or for optimizing the medication of an individual patient when other sources of evidence or the product information are neither available nor sufficient [11]. FAERs database is updated quarterly and includes patient demographics, academic information, medications, related AEs, and data sources. Re-ports of withdrawal-related AEs up to 2022 were imported into Microsoft Excel for statistical analyses. The classification and standardization of AEs in the FAERS data are made in accordance with MedDRA. Each report in the FAERS database is coded using preferred terms (PTs) of MedDRA terminology; a given PT can be assigned to the primary SOC in MedDRA. In addition, we collected clinical characteristics (sex, age, reporting year, reporting region, route of administration) of patients.

#### AEs signal detection

Currently, two types of methods are often used for AEs signal mining: frequency-based methods or Bayesian methods. And in this type of study, frequency-based methods, evaluating the reporting odds ratio (ROR) and proportional reporting rate (PRR) for signal detection ground are particularly common. Proportional reporting rate (PRR method) is the ratio of the ratio of adverse events (ADE) with exposure to a drug to the ratio of adverse events without exposure to that drug to determine the incidence of ADE for a drug at 95% confidence interval. The reporting probability (ROR) method is the ratio of the AE ratio for a particular adverse event (AE) in the presence of a drug to the AE ratio without exposure to that drug. Both methods are based on a four-grid table with simple calculations and good consistency of results (see Tables S1 and S2 in the Supplementary Information for the specific algorithm), so this method was chosen to complete this study. The information collected from the FAERS database was compared with the AEs listed in the drug instructions, and the corresponding PRR signals were ranked.

### Meta-analysis

#### Search strategy and selection criteria

Keywords were identified based on experimental design and subsequently searched by finding all relevant terms in PubMed under the mesh subject headings. The search strategy included as many eligible clinical trials as possible by permutating and combining all the obtained mesh terms “bevacizumab”, “vasic”, “Avastin”, “adverse reactions”, “adverse events”, and “clinical trials”. PubMed is a free MEDLINE database that provides literature search services in the biomedical and health sciences. MEDLINE is one of the most authoritative abstract-based medical literature databases in the world today. The Cochrane Central Register of Controlled Trials (CENTRAL) is a highly concentrated source of reports of randomized and quasi-randomized controlled trials. Created from multiple sources, CENTRAL is a single searchable source for high-quality evidence. Most CENTRAL records are taken from bibliographic databases (mainly PubMed and Embase.com), but records are also derived from other published and unpublished sources, including CINAHL, ClinicalTrials.gov and the WHO's International Clinical Trials Registry Platform. A systematic literature search was conducted using PubMed and the Cochrane Central Register of Controlled Trials (CENTRAL) up to 2022.

The inclusion criteria for the RCTs were as follows: 1) they included patients suffering from diseases for which bevacizumab is indicated, such as NSCLC, colorectal cancer, and retinal disease, etc.; 2) participants were assigned to treatment with bevacizumab (alone or in combination) or control (placebo or targeted therapy combination without bevacizumab); 3) the reported data included adverse events; 4) they included assessment of safety outcomes at any level. Non-randomized trials, RCTs without available reports on safety outcomes, comprising unclear drug interactions, and in which both arms of treatment ever received bevacizumab were excluded.

#### Data extraction and quality assessment

The following data were extracted by two reviewers independently: 1) basic in-formation about the selected studies: the first author's last name, year of publication, clinical trial number and phase, area of patients enrolled, number of patients in the treatment and control arms, median age, sex, and the indication for which bevacizumab was prescribed; 2) outcome indicators: number of all-grade adverse events. For the RCT studies that met the inclusion criteria, the full text, as well as supplementary data, were read to count various types of adverse events, as well as to identify and remove duplicate data based on the RCT experimental design and reporting of results. In addition, we searched references and conference proceedings included in the studies to supplement and obtain relevant materials. Any disagreements were resolved by a third reviewer. The guideline utilized is PRISMA, and registration ID is CRD42022380569.

The Cochrane Collaboration tool was used to assess the quality of the included studies, according to the following: 1) random sequence generation (selection bias), 2) allocation concealment (selection bias), 3) blinding of participants and personnel (performance bias), 4) blinding of outcome assessment (detection bias), 5) incomplete outcome data (attrition bias), 6) selective reporting (reporting bias), and 7) other bias. Two reviewers independently extracted the data according to the specified selection criteria. Differences in opinion were resolved through discussion with a third evaluator. Two investigators evaluated the included studies separately and the other resolved differences. The Cochrane Collaboration tool was used to assess the quality of the included studies [12].

#### Synthesis of results and statistical analysis

The results of our meta-analysis were evaluated using the Review Manager 5.4 system to enter data and perform statistical analysis.Relative Risk Ratio (RR) was used when the literature variables were dichotomous, and Standard Mean Difference (SMD) was used when the literature variables were continuous, both of which were expressed with 95% confidence intervals. The chi-square test was used in this study to evaluate the heterogeneity of the experimental and control groups, and the difference was statistically significant if *P* < 0.05. The chi-square test is the degree of deviation between the actual observed value of the statistical sample and the theoretical inferred value. The degree of deviation between the actual observed value and the theoretical inferred value determines the size of the chi-square value; if the chi-square value is larger, the greater the degree of deviation between the two; conversely, the smaller the deviation between the two; if the two values are exactly equal, the chi-square value is 0, indicating that the theoretical value is exactly the same.

Consistency or inconsistency between studies can be expressed in terms of heterogeneity, and commonly used indicators of heterogeneity include the Q statistic and the I2 statistic. the Q statistic is the sum of standardized weighted variances across studies, and a small *P* value (usually at the level of α < 0.10) indicates the presence of heterogeneity. However, the Q statistic has a high statistical power when the number of included studies is high.Therefore, in this study, the I statistic was used to assess the heterogeneity among the included studies. The I statistic is the proportion of observed between-study variation (due to true heterogeneity and not observed by chance). The formula was calculated as I2 = 100% × (Q—df)/Q. where Q is Cochran's Q heterogeneity statistic; df is the degree of freedom. Since all negative values of I2 are considered to be zero, the value of I2 is considered to be between 0 and 100%. i2 at 0%- 50% is usually considered to have no significant heterogeneity and a fixed effects model is used, while at 50%-100% a random effects model is used. A sensitivity analysis of the results was also performed to ensure the robustness of the results. The most common method for identifying publication bias is the funnel plot method, which is a scatter plot of sample content (or the inverse of the standard error of effect) versus the effect size (or the logarithm of the effect size), which can be RR, OR, RD, and death ratio or their logarithmic values. The funnel plot is based on the assumption that the precision of the effect size estimates increases with the sample size, and its width gradually becomes narrower with the increase in precision and finally tends to be point-like, and its shape resembles a symmetrical inverted funnel, so it is called a funnel plot. In other words, studies with small sample sizes, which have high numbers and low precision, are distributed at the bottom of the funnel plot in a symmetric arrangement; studies with large sample sizes, which have high precision, are distributed at the top of the funnel plot and concentrated in the middle. The funnel plot can be used to directly observe whether the effect size estimates of the original study are related to its sample size. However, when there is publication bias, the funnel plot appears asymmetrical and skewed. In addition to the use of funnel plot to detect publication bias, commonly used methods include rank correlation analysis, regression analysis, and cut-and-patch method. Among all the methods to identify publication bias, the funnel plot method is the most simple and practical, which can visually determine whether the effect size estimates are related to the sample size and determine whether there is publication bias by observing the symmetry of the scatter plot distribution.

Sensitivity analysis refers to the observation of differences in point estimates and interval estimates of combined values of effects when different models are used when low-quality literature is removed from the included literature according to study quality evaluation criteria when included studies are analyzed stratified according to sample size, and when inclusion and exclusion criteria are changed, Meta-analysis is re-run to examine whether there is any change in the conclusions. The aim was not to screen for the most favorable results but to examine the stability of the findings. When the sensitivity analysis results are consistent with the main analysis results, this indicates that the current conclusions are robust and less likely to be shaken. When the results of the sensitivity analysis are inconsistent with the results of the primary analysis, this indicates that the results of the primary analysis are not robust and the reasons for this need to be analyzed. After extracting the adverse events, data were encoded using the SOC list within the Medical Dictionary for Regulatory Activities (MedDRA). Data were sorted according to the SOC type and counted for each trial.

## Results

### Pharmacovigilance study

#### Descriptional analysis

**Fig. 1:**
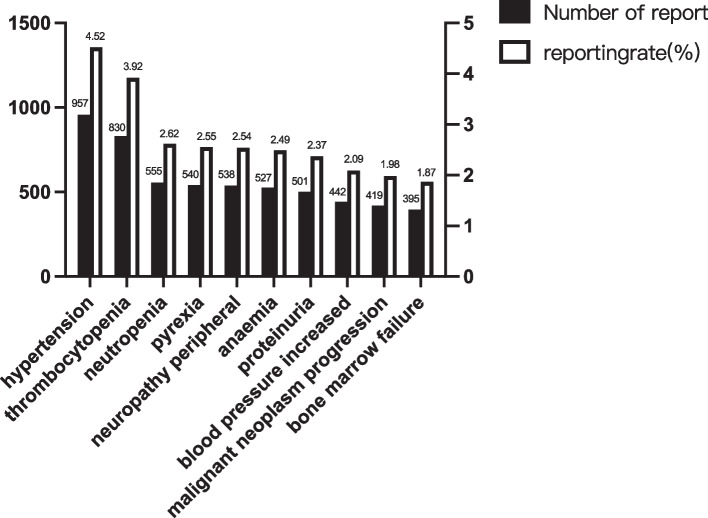
The histogram of the top 10 adverse reactions of bevacizumab in the FAERS database (detail number and rate were listed in Table S5)

A total of 21,161 adverse events related to bevacizumab were identified in the FAERS database up to Q2 2022. Among the affected patients, the proportions of men and women were equal. There was a large lack of age and administration data. Based on the cases where the information was provided, the median age ranged between 50 and 75 years, and the main route of administration was intravenous drip, as shown in Table 1. The total number of adverse events reported in China was 1767, accounting for 8.4%, as shown in Table S3. As the use of bevacizumab in pediatric patients is also in-creasing, more detailed baseline information has been compiled for pediatric patients. The top 3 AEs in the order of occurrence were hypertension, thrombocytopenia, and neutropenia. The most frequently reported adverse reactions were hypertension, thrombocytopenia, neutropenia, pyrexia, peripheral neuropathy, anemia, and proteinuria (Fig. 1) (more details in Table S4). And the top 10 AEs in pediatric patients off label use, intentional product use issue, drug ineffective, product use in unapproved indication malignant neoplasm progression no adverse event aspartate aminotransferase increased neoplasm progression, optic glioma, visual impairment (Tables S5, S6 and S7).

**Table 1:** The baseline of reports in FAERS

Medication information classification	Number of reports	Constituent radio %
Year		
2017	4520	21.35903979
2018	3492	16.50127587
2019	3353	15.84443814
2020	2674	12.63585672
2021	5827	27.53520461
2022	1295	6.119459408
Administration		
Intravenous drip	4649	21.968623
Intraocular	384	1.814573292
Ophthalmic	82	0.387487005
Others	112	0.529250543
Unknown	15,099	71.34958889
Gender		
Female	8445	39.90643606
Male	6831	32.2795577
UNK	5885	27.80928079
Age(year)		
< 18	222	1.049050184
【18, 50】	2029	9.587940648
(50, 75】	7131	33.69719308
> 75	1739	8.217559777
null	10,040	47.44353086

#### Signal of standardized MedDRA queries

The AEs signals detected using the ROR and PRR methods were counted and analyzed using MedDRA (Table 2, more details in Table S8), in which the highest PRR signal values are (all more than 300) acute peeking skin syndrome (PRR = 2926), stomal variables (PRR = 549). For the specific population of pediatric patients, the number of signals for adverse events was concentrated in Neoplasms benign, malignant and unspecified (incl cysts and polyps), Infections and infestations, and Investigations (Table S9). and the highest signals for adverse events were, in order, off-label use intentional product use issue drug ineffective product use in unapproved indications and so on.

**Table 2:** Detection results of the main safety signals of bevacizumab

Adverse event	PRR	ROR(95%CI)	Number of report
Vascular disorders			
Periphlebitis	108.375	108.395(33.378, 352.0159)	4
Varicose vein ruptured	41.407	41.425(20.432,, 83.98553)	9
Skin and subcutaneous tissue disorders			
Melanoderma	76.2	76.237(37.474, 155.09632)	5
Respiratory, thoracic and mediastinal disorders			
Laryngeal necrosis	365.765	365.868(103.237, 1296.6254)	6
Acquired tracheo-oesophageal fistula	182.882	182.934(63.467, 527.2818)	6
Dysaesthesia pharynx	156.756	156.823(67.871, 362.35614)	9
Renal and urinary disorders			
Ureteric fistula	406.406	406.501(97.139, 1701.1043)	5
Glomerular vascular disorder	365.765	366.283(207.98, 645.07720)	30
Nervous system disorders			
Secondary cerebellar degeneration	58.1	58.071(21.895, 154.02121)	5
Neoplasms benign, malignant and unspecified (incl cysts and polyps)			
Malignant glioma	171	170.746(64.987, 448.61810)	7
Optic glioma	155	155.275(79.438, 303.51222)	14
Metabolism and nutrition disorders			
Hyperamylasaemia	11.2	11.213(4.115, 30.55387)	4
Investigations			
Transaminases	112.543	112.575(42.784, 296.20913)	6
Prothrombin level decreased	54.865	54.911(32.924, 91.57980)	18
Protein urine	48.057	48.117(31.84, 72.715137)	27
Protein urine present	47.01	47.314(39.438, 56.763721)	190
kL-6 increased	46.446	46.455(15.945, 135.34621)	4
Injury, poisoning and procedural complications			
Stomal varices	549	548.88(169.015, 1782.4994)	9
Anastomotic fistula	457	457.53(193.96, 1079.2618)	15
Infections and infestations			
Suspected transmission of an infectious agent via product	277	278.447(215.665, 359.506111)	190
Bacterial endophthalmitis	106	106.068(50.478, 222.87923)	10
Hepatobiliary disorders			
Hepatic atrophy	128	127.925(83.936, 194.96963)	33
General disorders and administration site conditions			
Acral peeling skin syndrome	2926.12	2927.779(380.666, 22,518.1481)	12
Radiation interaction	121.922	121.95(41.679, 356.82110)	5
Perforation	58.465	58.577(41.648, 82.388171)	41
Gastrointestinal disorders			
Malignant gastrointestinal obstruction	97.537	97.565(37.851, 251.48515)	6
Gastrointestinal perforation	90.464	91.503(78.927,106.083655)	243
Rectourethral fistula	87.087	87.107(31.372, 241.86214)	5
Tongue geographic	53.341	53.358(23.549,120.90132)	7
Peritoneal disorder	48.03	48.059(26.51,87.12266)	13
Eye disorders			
Ciliary hyperaemia	243.843	244.108(136.931, 435.17123)	23
Retinal pigment epithelial tear	131.02	131.242(87.513, 196.82167)	36
Blood and lymphatic system disorders			
Splenic artery thrombosis	94.828	94.859(39.616, 227.13618)	7
Intravascular haemolysis	45.285	45.312(25.065, 81.91470)	13
Microangiopathic haemolytic anaemia	22.168	22.182(12.832, 38.342154)	14
Myelosuppression	20.619	20.922(18.658,23.4623808)	322
Bone marrow failure	19.597	19.95(17.991, 22.1244915)	395
Hypersplenism	19.05	19.055(7.668, 47.34964)	5
Surgical and medical procedures			
Portal vein embolisation	244	243.901(70.603, 842.5625)	71
Product issues			
Product contamination microbial	22.5	22.546(13.716, 37.062184)	17
Endocrine disorders			
Primary adrenal insufficiency	39.3	39.339(15.295, 101.17931)	5
Congenital, familial and genetic disorders			
bRCA2 gene mutation	65	65.037(21.583, 195.97615)	4
Cardiac disorders			
Cardiac ventricular thrombosis	11.6	11.572(6.484, 20.651253)	12

### Meta-analysis

#### Characteristics and quality of studies included in the meta-analysis

Our screening led to the identification of 356 articles. After the duplicate articles were removed and after filtering the articles based on their title and abstract, 19 articles remained. After full-text analysis and evaluation and applying the above-mentioned inclusion and exclusion criteria, we excluded 11 studies. Lastly, we included eight studies of high-quality [13–20] for systematic evaluation and meta-analysis. The literature screening process is illustrated in Figure S1 and the results of literature quality evaluation are shown in Figure S2. The characteristics of the eight included studies are shown in Table 3.

**Table 3:** Basic characteristics of included study

Author	Year	Clinical trial number	phase	Number of experimental groups	Number of control group	Indication	Countris/Area	Age, median	Gender, male (%)
David A. Reardon	2020	NCT02017717	3	185	184	Glioblastoma	12 countries	55 (22–76)	119 (64.3)
Chiara Cremolini	2018	NCT02295930	2	69	74	Colorectal cancer	IT	59 (53‐67)	42 (74)
Ingrid U Scott	2017	NCT01969708	3	182	180	Macular edema	US	69 (15verage)	107 (58.8)
Cesare Gridelli	2018	NCT01351415	3	243	232	NSCLC	several countries	63 (26–84)	155 (63.3)
Hiroaki Akamatsu	2020	UMIN000023761	2	40	41	NSCLC	unknown	68 (41–82)	31 (47)
Marla Lipsyc-Sharf	2022	NCT02292758	2	19	17	Colorectal cancer	US	55 (48–65)	20 (55.6)
Sermsiri Sangroongruangsri	2018	TCTR20141002001	3	5975	379	Retinal disease	THA	58 (average)	3000 (47.2)
Matthew H Kulke	2022	NCT01229943	2	75	75	pNETS	unknown	56 (21–86)	84 (56)

#### Results of the safety meta-analysis

**Fig. 2:**
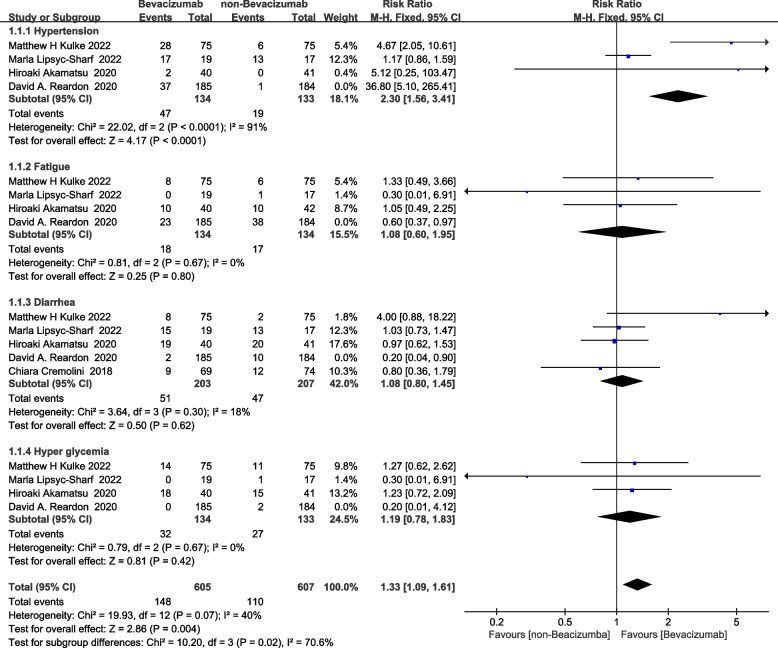
Forest plot of adverse reactions

The eight studies included in the meta-analysis provided data on bevacizumab-related adverse reactions of any severity. We identified hypertension, fatigue, diarrhea, and hyperglycemia as the AEs with the highest incidence, and selected them for heterogeneity testing and sensitivity analysis. The meta-analysis resulted in an RR of 1.33 (95% CI: 1.09–1.61, *p* = 0.004, I2 = 40%), indicating a higher risk of adverse effects, mainly hypertension, in the bevacizumab group than in the non-bevacizumab group (Fig. 2). The sample size was sufficient (n ≥ 2 included studies) for a meta-analysis to be meaningful. Therefore, after pooling the adverse reactions from all included studies, the most frequent ones, hypertension, diarrhea, fatigue, and hyperglycemia, were selected as the target outcomes for the meta-analysis. At the same time, a review of the drug inserts revealed that these four adverse events were ranked as the most common adverse reactions to bevacizumab, again indicating the reasonableness of the selection. In this study, the I statistic was used to assess the heterogeneity among the included studies. It is calculated as I2 = 100% × (Q—df)/Q. where Q is Cochran's Q heterogeneity statistic; df is the degree of freedom. Sensitivity analysis refers to the observation of differences in point estimates and interval estimates of combined values of effects when different models are used when low-quality literature is removed from the included literature according to study quality evaluation criteria when included studies are analyzed stratified according to sample size, and when inclusion and exclusion criteria are changed, Meta-analysis is re-run to examine whether there is any change in the conclusions. The funnel plot was symmetrical, indicating no significant publication bias (Figure S3). Clinical trials explore the efficacy and safety of drugs under standardized conditions, and the reliability of the results is high. However, randomized clinical trials are extremely limited in sample size and duration of use and cannot fully reflect and describe the safety of a drug. Therefore, there is a greater need for post-marketing surveillance of drugs. The safety of drugs is assessed through a combination of real-world pharmacovigilance and RCT study analysis, each with its own focus.

#### Adverse events at the SOC level in RCTs

Based on the classification and data statistics of each detailed adverse event, the bevacizumab-related adverse reactions with the highest incidence classified by SOC level after coding using MedDRA were: vascular disorders, investigations, general disorders and administration site conditions, metabolism and nutritional disorders, blood and lymphatic system disorders, gastrointestinal disorders, skin and subcutaneous tissue, respiratory, thoracic, and mediastinal disorders (Table 4).

**Table 4:** Top 8 system adverse reactions in RCTs

SOC	David A. Reardon (all)		Chiara Cremolini (> third grade)		Ingrid U Scott		Cesare Gridelli		Hiroaki Akamatsu		Marla Lipsyc-Sharf		Sermsiri Sangroongruangsri		Matthew H Kulke		Summary
	Bev	Non-Bev	Bev	Non-Bev	Bev	Non-Bev	Bev	Non-Bev	Bev	Non-Bev	Bev	Non-Bev	Bev	Non-Bev	Bev	Non-Bev	
Vascular disorders	37	1	2	1	0	1	54	28	2	0	17	14	250	3	29	6	445
Investigations	2	20							142	134	14	12			1	0	325
General disorders and administration site conditions	23	38	5	6			79	62	10	10	0	1			8	6	248
Metabolism and nutritional disorders	0	2					28	20	55	65	17	14			22	12	235
Blood and lymphatic system disorders			20	19			74	70	13	27			13	11	12	7	226
Gastrointestinal disorders	11	7	13	18			35	34	19	20	19	18	6	0	10	3	213
Skin and subcutaneous tissue	6	23	7	11			17		15	14	31	30					154
Respiratory, thoracic and mediastinal disorders	6	4					38	49	11	10			1	0			119

### Comparative analysis of AEs

AEs information obtained from RCT and FAERS was coded using MedDRA and sorted. The top ten adverse reactions overlapping in the RCT and FAERS belonged to the following SOC levels: vascular disorders; general disorders and administration site conditions; blood and lymphatic system disorders; gastrointestinal disorders; and respiratory, thoracic, and mediastinal disorders (Table S10). The PRR signals of frequent ad-verse reactions listed in the drug specifications are presented in Table 5 Our comparative analysis revealed the occurrence of various rare adverse reactions which are not listed in the drug specifications, such as nasal septum perforation, gallbladder perforation, gastrointestinal ulcer, and mandibular necrosis. These reactions had a PRR signal higher than 2 (*p* < 0.05; Table 6).

**Table 5:** Expression of adverse effects in instructions in FAERs

SOC	AEs	PRR
Vascular disorders	hypertension	6.505
	Arterial thromboembolism	6.753
	Deep Vein Thrombosis	3.548
	Hemorrhage	2.66
Skin and subcutaneous tissue disorders	Hand-foot syndrome	11.657
Respiratory, thoracic and mediastinal disorders	pulmonary infarction	4.653
	epistaxis	5.028
Renal and urinary disorders	proteinuria	37.155
Metabolism and nutrition disorders	decreased appetite	2.213
	hypomagnesaemia	3.993
Blood and lymphatic system disorders	neutropenia	4.651
	anaemia	4.05
	thrombocytopenia	5.357
	febrile neutropenia	6.758
	leukopenia	4.68
	lymphopenia	2.062
General disorders and administration site conditions	pyrexia	2.342
	mucosal inflammation	7.727
Gastrointestinal disorders	gastrointestinal perforation	90.464
	intestinal obstruction	9.696
	stomatitis	4.656
	rectal hemorrhage	2.439
	gastrointestinal disorder	2.75
Cardiac disorders	cardiac failure	2.029
Nervous system disorders	peripheral sensory neuropathy	21.777

**Table 6:** Expression of rare adverse reactions in instructions in FAERs

SOC	AEs	PRR
Respiratory, thoracic and mediastinal disorders	nasal septum perforation	47.502
Musculoskeletal and connective tissue disorders	osteonecrosis of jaw	3.999
Nervous system disorders	posterior reversible encephalopathy syndrome	9.279
	hypertensive encephalopathy	18.288
Infections and infestations	necrotising fasciitis	20.261
Hepatobiliary disorders	gallbladder rupture	14.438
	gastric ulcer	2.21
Gastrointestinal disorders	duodenal ulcer	3.706

## Discussions

FAERS is a useful tool for the FDA to look for new safety issues that may be associated with marketed products, to assess manufacturers' compliance with reporting regulations, and to respond to outside requests for information. reports in FAERS are evaluated by clinical reviewers at the Center for Drug Evaluation and Research (CDER) and the Center for Biologics Evaluation and Research (CBER) to monitor products after they have been FDA-approved for safety. If a potential safety issue is identified in FAERS, further evaluation is conducted. Further evaluation may include studies using other large databases, such as those in the Sentinel System. Based on the assessment of a potential safety concern, FDA may take regulatory action to improve the safety of the product and protect public health, such as updating the product's labeling information, restricting the use of the drug, communicating new safety information to the public, or, in rare cases, removing the product from the market.

### Comparison with similar studies

Bevacizumab has been shown to have significant efficacy against cancer progression and retinal disease in previous studies, and it is often unanticipated adverse effects of various kinds that have led to the suspension or even discontinuation of the drug. Previous concerns surrounding bevacizumab stem from various reports on an in-creased risk of serious and even fatal bleeding observed in early randomized trials. Various safety signals were detected in subsequent trials involving anti-angiogenic drugs [21–23]. This is because VEGF is essential not only for physiological and pathological angiogenesis but also for the maintenance of vascular homeostasis. Its pharmacological blockade may lead to endothelial dysfunction and adverse vascular effects such as venous thromboembolism [24].In a previous study, the renal biopsies of most patients with bevacizumab-related proteinuria showed renal thrombotic microangiopathy [25] and transient proteinuria [26]. The most frequent adverse reactions associated with bevacizumab included hypertension (32%), bleeding and thrombotic events (16%), proteinuria, headache, rash, and dyspnea [27, 28]. Bevacizumab has a “black box” warning for gastrointestinal perforation with lethal potential [29]. Bevacizumab was approved for entry into the Chinese market in 2017, further expanding the user base. Further comprehensive evaluation of the safety of this drug is indispensable for clinical use and will facilitate further prevention and control of related adverse events.

### Key findings

In the FAERS database, the adverse reaction signals for bevacizumab comprised gastrointestinal disorders, benign, malignant, and unspecified neoplasms (including cysts and polyps), eye disorders, infections, and infestations, as well as others. This indicates a broad spectrum of adverse reactions of bevacizumab, which is consistent with its directions. However, compared to the specifications, we found a higher incidence of thrombocytopenia, neutropenia, and peripheral febrile neuropathy, and a lower incidence of fatigue, diarrhea, and abdominal pain. Both our meta-analysis as well as FAERS point out hypertension as the most frequent adverse effect. Therapy with bevacizumab may need particular monitoring for AEs such as hypertension, listed in the drug specifications and detected with high frequency in pharmacovigilance data. VEGF contributes to the regulation of blood pressure [30]. Direct administration of VEGF induces vasodilation and lowers blood pressure [31–33], while topical administration of bevacizumab rapidly reduces endothelium-dependent vasodilation in hu-man individuals [30]. Subsequently, VEGF expression in renal endothelial cells and podocytes is required for the maintenance of normal glomerular structure and filtration [34]. Characteristic thrombotic microangiopathy has been observed in patients treated with bevacizumab, which may be a cause of glomerular injury and elevated blood pressure [35]. Thus, hypertension might be related to renal insufficiency. In this meta-analysis, we assessed the safety of bevacizumab by analyzing the adverse reactions associated with bevacizumab reported in RCTs over the past 5 years. Treatment with bevacizumab may require specific monitoring for AEs such as hypertension, which are listed in the drug insert and detected with high frequency in pharmacovigilance data. SOC coding classification of adverse events associated with bevacizumab using MedDRA revealed a major focus on vascular disease, investigations, general disease and administration site conditions, metabolic and nutritional disorders.

Bevacizumab specifications list hypertensive encephalopathy, necrotizing fasciitis and nasal septum perforation as very rare (PRR = 18.288), rare (PRR = 20.261), and un-known (PRR = 47.502), respectively. But adverse reaction signal mining using real-world data revealed high-intensity signals for these adverse reactions, which hints that they should be clinically monitored. Early prevention and timely symptomatic management can be effective in preventing serious ad-verse events.

### Interpretation of findings

Blood vessels in the nasal septum are scarce. Furthermore, owing to its anti-angiogenic effect, bevacizumab alters these scarce blood vessels impairing the viability of the tissue in the nasal septum and leading to perforation [36]. Besides nasal septal perforation, bevacizumab may be associated with widespread sinus toxicity. D’amico et al. investigated bevacizumab-related sinus toxicity and found mild rhinorrhea and sinus irritation were the most frequent. The underlying mechanism is not clear and may be multifactorial [37]. Inhibition of VEGF-A results in reduced angiogenesis, mucositis, and poor wound healing which may all contribute to sinus toxicity [36–38]. Other non-related contributing factors include nerve damage, immunosuppression, and trauma. In most cases, conservative treatment with local moisturizer is adequate because the severity of the disease is minimal [36, 37, 39]. Bevacizumab-induced nasal perforation tends to not progress over time, although no long-term studies exist. Given the high incidence of sinus mucositis, oncologists may want to consider the prophylactic initiation of topical nasal moisturizing when starting therapy with bevacizumab.

Necrotizing fasciitis has been reported in patients treated with bevacizumab, usually secondary to wound healing complications, gastrointestinal perforation, or fistula formation [40]. Necrotizing fasciitis is a rare but life-threatening infection of the soft tissue characterized by rapidly spreading necrosis of the superficial fascia and subcutaneous tissues. Immunocompromised and diabetic patients are at higher risk of developing necrotizing fasciitis than the general population [41]. The combined pro-thrombotic and anti-angiogenic effects of bevacizumab cause tissue ischemia and necrosis [42], and are also associated with poor wound healing. This results in increased wound susceptibility to bacterial infections [43–45]. All these factors contribute to the occurrence of necrotizing fasciitis. Early recognition and discontinuation of therapy with bevacizumab are essential to managing this complication which may be life-threatening, especially in patients who are already immunosuppressed. In patients with moderate-to-severe proteinuria and uncontrolled hypertension, bevacizumab can be temporarily discontinued until clinical stabilization. However, if a patient has gastrointestinal perforation, severe bleeding, thromboembolism, necrotizing fasciitis, or hypertensive encephalopathy, treatment should be permanently discontinued.

### Limitations and potential future research directions

The present study has several limitations. First, the meta-analysis involved only eight randomized controlled trials with a limited number of participants. The trials had different designs, using different regimens and doses of bevacizumab, prescribed for various indications, and having distinct control groups. This led to increased heterogeneity among the RCTs. Second, the number of RCTs specifically designed to assess the safety of drug use is small, focusing mainly on efficacy and including limited data. Third, FAERS is a spontaneous reporting system with partial reporting bias and missing data.The information in FAERS changes daily and the number of cases may increase or decrease. Therefore, information obtained from the website may also change over time. Many factors (e.g. product launch cycle, region, and underreporting) can affect case reporting, which can bias the initial safety assessment, such as overlooking the safety of some common adverse reactions. Fourth, all signal detection results only indicate a statistical correlation be-tween the administration of bevacizumab and the occurrence of an adverse reaction. The existence of a true causal relationship should be further confirmed. Finally, we have identified adverse reactions of bevacizumab increasing the knowledge on its safe-ty profile. The next step we will have a mind to attempt to identify the specific risk factors contributing to their development.

As a next step, it is expected that additional clinical data will be collected and stratified according to criteria such as the indication for bevacizumab, mode of administration and patients' co-morbidities to further explore risk factors associated with adverse events, which may be a potential research direction to provide more detailed clinical guidance.

## Conclusion

This study used both safety reports originating from the FARES database and RCTs for a systematic evaluation of the adverse effects of bevacizumab. A true trend of the AEs of bevacizumab was obtained by comparison with its specifications. The meta-analysis and FAERS both noted that hypertension was the most common adverse reaction. Bevacizumab treatment may require special monitoring for adverse reactions such as hypertension, which are listed in the drug insert and frequently detected in pharmacovigilance data. In addition, this study also showed a high signal value for the correlation between bevacizumab use and the occurrence of rare adverse reactions such as hypertensive encephalopathy, necrotising fasciitis and nasal septal perforation. Thus, in clinical practice, those at high risk for these reactions should be monitored, and the medication should be adjusted promptly.

### Supplementary Information

**Additional file 1: Table S1.** The region of reports in FAERS. **Table S2.** The baseline of reports of pediatric patients in FAERS. **Table S3.** The region of reports of pediatric patients in FAERS. **Table S4.** Top 10 number of AEs reports of pediatric patients in FAERS. **Table S5.** Top 50 number of AEs reports in FAERS, which all are greater than or equal to 190. **Table S6.** Top 10 number of AEs reports in FAERS and rates. **Table S7.** Detection results of the main safety signals of bevacizumab in pediatric patients. **Figure S1.** Literature screening process and results. **Figure S2.** The funnel plot. **Table S8.** AEs by system in the top 10 of both RCT and FAERS. **Table S9.** A “2X2” table for signal calculations. **Table S10.** The calculation formulas and standards for the ROR and PRR methods. **Figure S3.** Risk of bias graph.

## Data Availability

The original contributions presented in this study are included in the article/Supplementary Materials, and further inquiries can be directed to the corresponding author.
