# Supplementary material for: Five years of safety profile of bevacizumab: an analysis of real-world pharmacovigilance and randomized clinical trials
Source: J Pharm Health Care Sci. 2024 Jan 2;10:1. doi: 10.1186/s40780-023-00314-w (PMC10763108; doi:10.1186/s40780-023-00314-w)
Supplement: Supplementary file 1 — Additional file 1: Table S1. The region of reports in FAERS. Table S2. The baseline of reports of pediatric patients in FAERS. Table S3. The region of reports of pediatric patients in FAERS. Table S4. Top 10 number of AEs reports of pediatric patients in FAERS. Table S5. Top 50 number of AEs reports in FAERS, which all are greater than or equal to 190. Table S6. Top 10 number of AEs reports in FAERS and rates. Table S7. Detection results of the main safety signals of bevacizumab in pediatric patients. Figure S1. Literature screening process and results. Figure S2. The funnel plot. Table S8. AEs by system in the top 10 of both RCT and FAERS. Table S9. A “2X2” table for signal calculations. Table S10. The calculation formulas and standards for the ROR and PRR methods. Figure S3. Risk of bias graph. [file 40780_2023_314_MOESM1_ESM.docx]

Supplementary Material

**Five Years of Safety Profile of Bevacizumab: An Analysis of Real-World Pharmacovigilance and Randomized Clinical Trials**

**Li Wang ^1, 3, #^, Yibo Fei^4, #^, Han Qu^1, 2^, Haiyang Zhang^1, 2^, Yuanyuan Wang^1^，Zhenghua Wu ^1, 2, *^, Guorong Fan ^1, 2, *^**

*** Correspondence:** Guorong Fan: [fanguorong@sjtu.edu.cn](mailto:fanguorong@sjtu.edu.cn)

Zhenghua Wu: [zhenghua.wu@shgh.cn](mailto:zhenghua.wu@shgh.cn)

**Table.S1 The region of reports in FAERS**

| Area | Number of reports，（%） | Number of country | The country with the most reports（Number of reports） |
| --- | --- | --- | --- |
| North America | 9733,(45.9928173) | 7 | US(+PR)，(9119) |
| Asia | 6870,(32.4653883) | 27 | JP,（4264） |
|  |  |  | CN,(1767) |
| Europe | 3750,(17.7212797) | 32 | FR,(814） |
| South America | 322,(1.5215953) | 12 | BR,(144) |
| Africa | 189,(0.8931102) | 12 | EG,(44) |
| Oceania | 179,(0.8458558) | 2 | AU,(169) |
| COUNTRY NOT SPECIFIED | 118,(0.5576032) |  |  |

**Table S2. The baseline of reports of pediatric patients in FAERS**

| Route | Number of reports |
| --- | --- |
| Route |  |
| Intraocular | 5 |
| Intravenous drip | 29 |
| Irrigation | 1 |
| Ophthalmic | 1 |
| Oral | 28 |
| Other | 22 |
| Subcutaneous | 2 |
| Unknown | 134 |
| Gentle |  |
| Female | 106 |
| Male | 88 |
| Unknown | 28 |
| Age |  |
| 0 | 22 |
| 1 | 9 |
| 2 | 7 |
| 3 | 22 |
| 4 | 10 |
| 5 | 18 |
| 6 | 5 |
| 7 | 4 |
| 8 | 7 |
| 9 | 6 |
| 10 | 11 |
| 11 | 23 |
| 12 | 10 |
| 13 | 7 |
| 14 | 19 |
| 15 | 12 |
| 16 | 21 |
| 17 | 9 |
| Report time |  |
| 2017 | 38 |
| 2018 | 39 |
| 2019 | 34 |
| 2020 | 24 |
| 2021 | 73 |
| 2022 | 14 |

**Table S3. The region of reports of pediatric patients in FAERS**

| Area | Number of reports | Number of country | The country with the most reports（Number of reports） |
| --- | --- | --- | --- |
| Africa | 1 | 1 | ZA,(1) |
| Asia | 44 | 5 | JP,(35) |
| Europe | 45 | 9 | CZ,(12) |
| North America | 125 | 2 | US,(123) |
| Oceanica | 4 | 1 | AU,(4) |
| South America | 1 | 1 | BR,(1) |
| COUNTRY NOT SPECIFIED | 2 |  |  |

**Table S4. Top 10 number of AEs reports of pediatric patients in FAERS**

| Adverse events | Number of reports |
| --- | --- |
| off label use | 78 |
| intentional product use issue | 30 |
| drug ineffective | 16 |
| product use in unapproved indication | 16 |
| malignant neoplasm progression | 15 |
| no adverse event | 14 |
| Aspartame aminotransferase increased | 13 |
| neoplasm progression | 12 |
| optic glioma | 11 |

**Table. S5 Top 50 number of AEs reports in FAERS, which all are greater than or equal to 190.**

| Adverse Event | Number of reports |
| --- | --- |
| off label use | 2276 |
| intentional product use issue | 991 |
| hypertension | 957 |
| disease progression | 895 |
| no adverse event | 783 |
| neutropenia | 555 |
| pyrexia | 540 |
| neuropathy peripheral | 538 |
| anaemia | 527 |
| proteinuria | 501 |
| blood pressure increased | 442 |
| thrombocytopenia | 420 |
| malignant neoplasm progression | 419 |
| platelet count decreased | 410 |
| bone marrow failure | 395 |
| decreased appetite | 391 |
| endophthalmitis | 349 |
| myelosuppression | 322 |
| epistaxis | 316 |
| neoplasm progression | 315 |
| neutrophil count decreased | 300 |
| visual impairment | 277 |
| febrile neutropenia | 272 |
| blindness | 267 |
| white blood cell count decreased | 261 |
| Pal-mar-plantar erythrodysaesthesia syndrome | 256 |
| pulmonary embolism | 246 |
| gastrointestinal perforation | 243 |
| interstitial lung disease | 243 |
| intestinal obstruction | 237 |
| hemorrhage | 235 |
| ascites | 226 |
| stomatitis | 219 |
| renal impairment | 218 |
| hepatic function abnormal | 217 |
| suspected transmission of an infectious agent via product | 190 |
| protein urine present | 190 |
| intestinal perforation | 190 |
| metastases to lung | 190 |
| neurotoxicity | 190 |
| intercepted medication error | 190 |
| metastases to liver | 190 |
| mucosal inflammation | 190 |
| leukopenia | 190 |
| deep vein thrombosis | 190 |
| pleural effusion | 190 |
| gastrointestinal disorder | 190 |
| general physical health deterioration | 190 |
| sepsis | 190 |
| cardiac failure | 190 |

**Table.S6 Top 10 number of AEs reports in FAERS and rates.**

| Adverse Events | Number of reports | Reporting rate（%） |
| --- | --- | --- |
| hypertension | 957 | 4.522470583 |
| thrombocytopenia | 830 | 3.922309910 |
| neutropenia | 555 | 2.622749397 |
| pyrexia | 540 | 2.551864279 |
| neuropathy peripheral | 538 | 2.542412929 |
| anemia | 527 | 2.490430509 |
| proteinuria | 501 | 2.367562970 |
| blood pressure increased | 442 | 2.088748169 |
| malignant neoplasm progression | 419 | 1.980057653 |
| bone marrow failure | 395 | 1.866641463 |

**Table S7. Detection results of the main safety signals of bevacizumab in** **pediatric patients.**

| Adverse event | Number of reports | PRR | ROR（95%CI） |
| --- | --- | --- | --- |
| Blood and lymphatic system disorders |  |  |  |
| thrombocytopenia | 8 | 5.74 | 5.917（2.912，12.022） |
| myelosuppression | 6 | 11.681 | 11.978（5.283，27.156） |
| bone marrow failure | 4 | 6.426 | 6.526（2.415，17.631） |
| pancytopenia | 4 | 5.411 | 5.492（2.034，14.826） |
| Congenital, familial and genetic disorders |  |  |  |
| hypermutation | 4 | 833.231 | 848.502（188.786，3813.605） |
| Eye disorders |  |  |  |
| visual impairment | 11 | 17.671 | 18.54（10.031，34.269） |
| blindness | 6 | 24.036 | 24.675（10.8，56.377） |
| Gastrointestinal disorders |  |  |  |
| diarrhea | 8 | 2.26 | 2.307（1.138，4.679） |
| General disorders and administration site conditions |  |  |  |
| no adverse event | 14 | 4.129 | 4.339（2.522，7.468） |
| disease progression | 9 | 10.733 | 11.145（5.689，21.834） |
| death | 7 | 2.354 | 2.398（1.128，5.099） |
| drug effective for unapproved indication | 6 | 22.863 | 23.47（10.28，53.585） |
| Hepatobiliary disorders |  |  |  |
| hepatocellular injury | 5 | 12.253 | 12.513（5.113，30.624） |
| Immune system disorders |  |  |  |
| drug hypersensitivity | 4 | 2.671 | 2.701（1.003，7.277） |
| Infections and infestations |  |  |  |
| infection | 4 | 3.396 | 3.44（1.276，9.273） |
| Injury, poisoning and procedural complications |  |  |  |
| off label use | 78 | 3.714 | 5.184（3.933，6.833） |
| intentional product use issue | 30 | 23.852 | 27.423（18.546，40.548） |
| product administration error | 4 | 3.633 | 3.682（1.366，9.926） |
| aspartate aminotransferase increased | 13 | 16.546 | 17.513（9.931，30.881） |
| alanine aminotransferase increased | 10 | 10.278 | 10.716（5.655，20.307） |
| neutrophil count decreased | 8 | 13.122 | 13.575（6.655，27.692） |
| white blood cell count decreased | 7 | 9.428 | 9.702（4.545，20.71） |
| platelet count decreased | 6 | 6.544 | 6.698（2.964，15.136） |
| blood creatinine increased | 5 | 9.644 | 9.843（4.029，24.047） |
| lymphocyte count decreased | 4 | 13.367 | 13.594（5.005，36.927） |
| Metabolism and nutrition disorders |  |  |  |
| hyperglycaemia | 7 | 13.713 | 14.127（6.602，30.23） |
| hyperkalaemia | 6 | 23.145 | 23.76（10.405，54.257） |
| hyponatraemia | 4 | 7.598 | 7.719（2.854，20.874） |
| Neoplasms benign, malignant and unspecified (incl cysts and polyps) |  |  |  |
| malignant neoplasm progression | 15 | 26.257 | 28.088（16.462，47.923） |
| neoplasm progression | 12 | 41.204 | 43.501（23.883，79.235） |
| optic glioma | 11 | 491.011 | 516.557（231.833，1150.962） |
| astrocytoma | 10 | 312.462 | 327.153（151.33，707.257） |
| glioma | 5 | 111.593 | 114.142（43.667，298.36） |
| Nervous system disorders |  |  |  |
| cerebral ventricle dilatation | 6 | 69.436 | 71.337（30.37，167.563） |


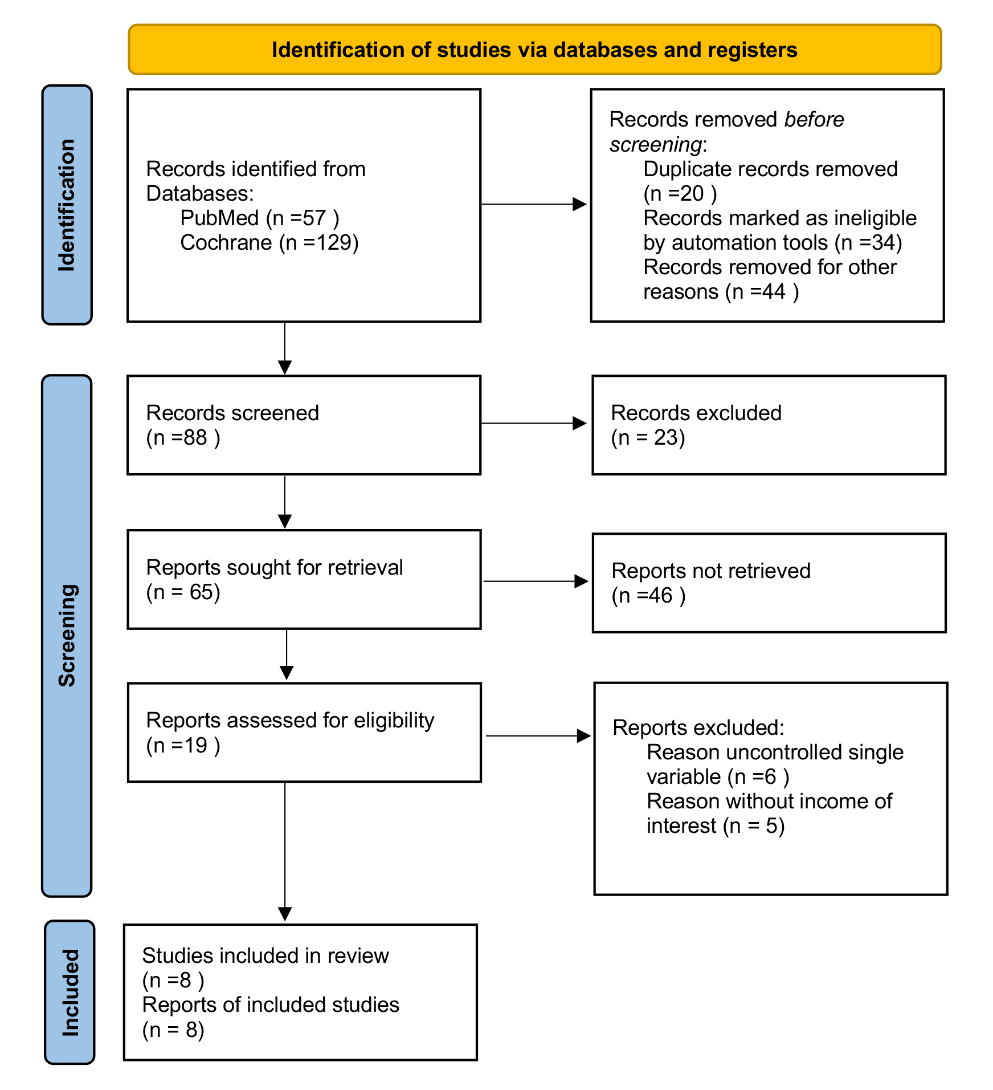


**Figure S1.** Literature screening process and results.


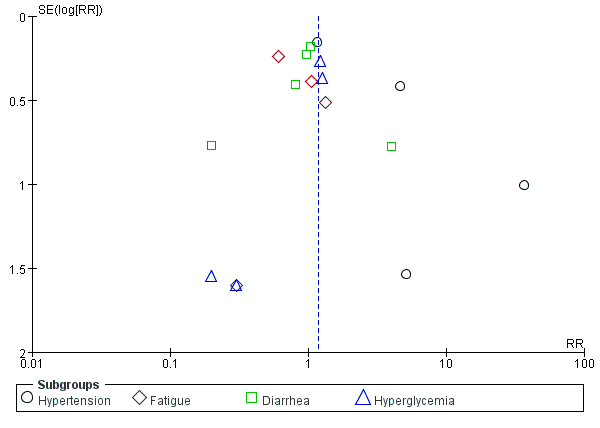


**Figure S2. The funnel plot**

**Table.S8 AEs by system in the top 10 of both RCT and FAERS**

| SOC | Number of reports in RCT | Number of reports in FAERS |
| --- | --- | --- |
| Vascular disorders | 445 | 1752 |
| General disorders and administration site conditions | 248 | 3329 |
| Blood and lymphatic system disorders | 226 | 3370 |
| Gastrointestinal disorders | 213 | 2752 |
| Respiratory, thoracic and mediastinal disorders | 119 | 1485 |

**Table S9. A “2X2” table for signal calculations**

| Type of drugs | Target ADR | Other ADR |
| --- | --- | --- |
| Target drugs | A | B |
| Other drugs | C | D |

**Table S10. The calculation formulas and standards for the ROR and PRR methods**

| Method to detect | Formula of computation | Criterion |
| --- | --- | --- |
| ROR | ROR=AD/(BC) | 95%lower limit CI＞1，A＞3 |
| PRR | PRR=A(C+D)/[C(A+B)] | PRR＞2，A＞3 |

**
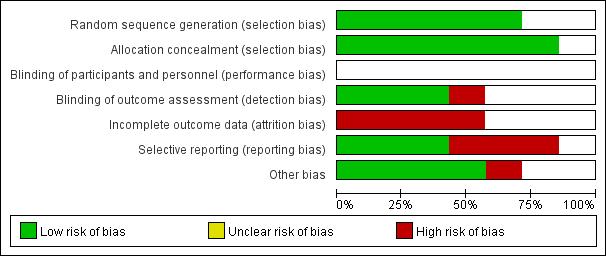

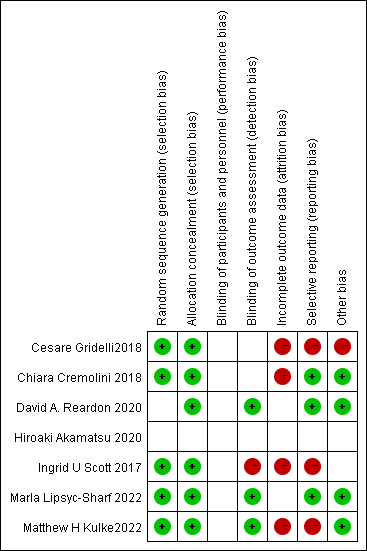
**

**Figure S3 Risk of bias graph**

.
